# Driving Nails

**Published:** 1867-12

**Authors:** 


### DRIVING NAILS

Within a year we have seen it stated, as a new truth, that if a nail were wetted in the mouth an if, in addition, the narrow edge was placed with the grain of the wood, it would seldom split the board into which it was driven. We well remember to have seen our father do this as far back as in eighteen hundred and eighteen. But

